# Targeting 3-phosphoinositide-dependent protein kinase 1 associated with drug-resistant renal cell carcinoma using new oridonin analogs

**DOI:** 10.1038/cddis.2017.121

**Published:** 2017-03-23

**Authors:** Jiancheng Zhou, Eun-Jin Yun, Wei Chen, Ye Ding, Kaijie Wu, Bin Wang, Chunyong Ding, Elizabeth Hernandez, John Santoyo, Rey-Chen Pong, Haiying Chen, Dalin He, Jia Zhou, Jer-Tsong Hsieh

**Affiliations:** 1 Department of Urology, Shaanxi Provincial People's Hospital, Xi'an 710068, Shaanxi, P.R. China; 2 Department of Urology, University of Texas Southwestern Medical Center, Dallas, TX 75390, USA; 3 Department of Urology, The First Affiliated Hospital, Medical School of Xi'an Jiaotong University, Xi'an 710061, China; 4 Department of Pharmacology and Toxicology, University of Texas Medical Branch, Galveston, TX 77555, USA; 5 Institute of Urology, Medical School of Xi'an Jiaotong University, Xi'an 710061, China

## Abstract

The current agents used for renal cell carcinoma (RCC) only exhibit the moderate response rate among patients. Development of drug resistance eventually fuels the need of either more potent drugs or new drugs to target the resistant pathways. Oridonin is a diterpenoid isolated from the Chinese medicinal herb *Rabdosia rubescens* and has been shown to have antitumor activities in many cancers. We previously developed new synthetic methodologies to modify structurally diversified diterpenoids and designed a series of nitrogen-enriched oridonin analogs. In this study, we screened a variety of oridonin analogs based on their cytotoxicity using MTT assay and identify the most potent candidate, namely, CYD-6-17. CYD-6-17 exhibited a high potency to inhibit the *in vitro* growth of several drug-resistant RCC cells as well as endothelial cells stimulated by tumor cells at nanomolar range. Delivery of CYD-6-17 significantly inhibited RCC tumor growth using xenograft model. Mechanistically, it targeted the 3-phosphoinositide-dependent protein kinase 1 gene that appeared to be a potent regulator of AKT and was associated with patient survival after targeted therapies. This offers a new rational therapeutic regimen of CYD-6-17 to drug-resistant RCC based on its novel mechanism of action.

Renal cell carcinoma (RCC) is the most common type of kidney cancer and extremely lethal urologic malignancy. Particularly, metastatic RCC (mRCC) resistant to many therapies leading to the poor prognosis represents a significant challenge for clinicians. Currently, small-molecular agents targeting mTOR/VEGF-signaling axis are the first-line therapy for mRCC.^1^ Despite of the initial efficacy, these agents only show moderate response rate among patients who develop drug resistance rapidly.^2^ Thus, the development of new agents to target resistant mechanism become an urgent need for mRCC therapy.

Herbal medicine has drawn vast attention to become potential new cancer therapeutics due to its remarkable growth inhibition, low side effects, and multiple actions that become less prone to develop drug resistance.^3^ Oridonin is a diterpenoid isolated from the Chinese medicinal herb *Rabdosia rubescens*, which is common remedy in China and Japan. It is traditionally used as an anti-microbial, anti-inflammatory, and anti-oxidant compound.^4, 5^ Despite the promising effects of oridonin, its clinical development has been hampered by its limited aqueous solubility and bioavailability.^6^ To solve this problem, many different approaches have been developed to modify its structure to increase the solubility and bioavailability of oridonin in order to increase its potency. Recently, we developed new methodologies to synthesize a series of nitrogen-enriched oridonin analogs.^6^

In the present study, we screened a serial of oridonin analogs to identify the most potent compound, CYD-6-17, which could significantly suppress the growth of drug-resistant RCC cells with different genetic profiles *in vitro* and exhibit *in vivo* efficacy. Although several molecular targets of oridonin have been characterized by other studies, our analyses delineated 3-phosphoinositide-dependent protein kinase 1 (PDPK1) gene as a new target of CYD-6-17. PDPK1 is able to activate AKT often highly active in drug-resistant RCC cells. We conclude that CYD-6-17 is a promising agent for drug-resistant RCC by inhibiting AKT activities.

## Results

### Screening of oridonin analogs for the growth inhibition of drug-resistant RCC cells

**Figure 1:**
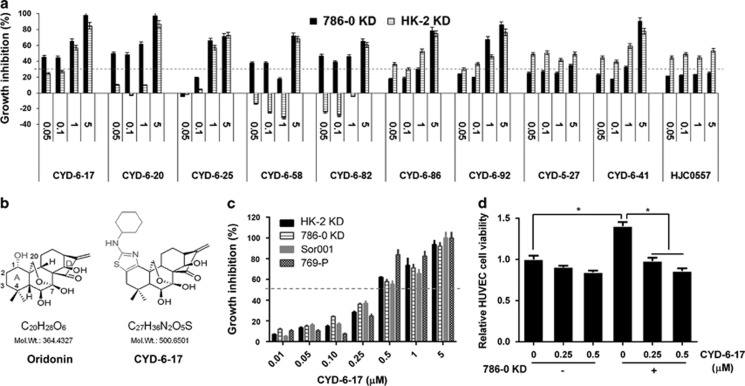
The effect of oridonin analogs on the growth of RCC cells. (**a**) The effect of oridonin analogs on the cell growth of HK-2 KD and 786-0 KD determined by MTT assay. The growth inhibition rate was calculated based on the control. (**b**) Chemical structure of oridonin and CYD-6-17. (**c**) The effect of CYD-6-17 on the growth of a variety of RCC cell lines determined by MTT assay. The growth inhibition rate was calculated based on the control. (**d**) The effect of CYD-6-17 on the growth of HUVEC alone or co-cultured with 786-0 KD cells determined by crystal violet assay with OD 555 nm. *P<0.05

Oridonin has been found to be an growth inhibitor in several types of tumor^7^ but the clinical development of oridonin has been hampered because of its relatively moderate potency, limited aqueous solubility, and bioavailability.^8^ Recently, we designed new chemical synthetic procedure to develop a series of nitrogen-enriched oridonin analogs.^6^ We also established several RCC cell lines (i.e., 786-0 KD and HK-2 KD) resistant to mTOR and tyrosine kinase inhibitors from our recent publication.^9^ Using these two models for initial screening of 10 oridonin analogs (Figure 1a), we identified CYD-6-17 that is fused at C-1 and C-2 of the A-ring as compared with oridonin (Figure 1b) with enhanced anticancer potency than oridonin (Supplementary Figure 1).

### High potency of CYD-6-17 in inhibiting the growth of RCC cell lines and endothelia induced by RCC

We therefore examined the effect of CYD-6-17 using a panel of RCC cells including drug resistant and sensitive RCC cell lines, the data (Figure 1c and Supplementary Figure 2C) indicated that CYD-6-17 showed the similar IC_50_ (around 500 nM range) for all RCC lines examined. It appeared that CYD-6-17 exhibited high potency in both parental and drug-resistant cells (Supplementary Figure 2B). It is known that tumor microenvironment such as vascular endothelia play a critical role in RCC development, which is considered as a key therapeutic target in current RCC therapeutic regimen such as tyrosine kinase inhibitor. We further determined whether CYD-6-17 is able to target tumor-induced vascular endothelia growth. As shown in Figure 1d, using both 786-0 KD and human umbilical vein endothelial cell (HUVEC) in a co-culture system, CYD-6-17 did not cause a significant toxicity on HUVEC alone. However, CYD-6-17 was able to inhibit 786-0 KD-induced growth of HUVEC cells, which also implied the specificity of CYD-6-17 on RCC cells.

### Effect of CYD-6-17 on RCC growth via apoptosis induction and cell cycle arrest

**Figure 2:**
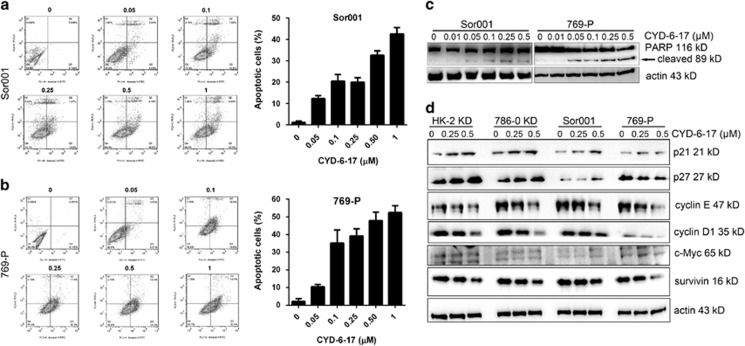
The induction of apoptosis and cell cycle arrest RCC cells by CYD-6-17. (**a** and **b**) The cell apoptosis of Sor001 and 769-P cells were determined 24 h after the indicated concentration of CYD-6-17 treatment using PI/Annexin V assay (left panel) and the quantification of cell apoptosis as depicted (right panel). (**c**) Western blot analyses of the expression of apoptotic markers (cleaved PAPR) in Sor001 and 769-P cells after CYD-6-17 treatment. (**d**) Western blot analyses of the expression of cell cycle regulators in RCC cells after CYD-6-17 treatment. Actin was used as loading control

To investigate the biological effect of CYD-6-17 on RCC cells, we determined whether CYD-6-17 was able to induce apoptosis in RCC cells. As shown in Figures 2a and b, the incremental concentration of CYD-6-17 induced cell apoptosis in a dose-dependent manner. Consistently, CYD-6-17 increased cleaved PARP levels, a biochemical marker of apoptosis, in a dose-dependent manner (Figure 2c). Meanwhile, CYD-6-17 was shown to inhibit the expression of cyclin protein (i.e., cyclin E and D) but increase the expression of cyclin-dependent kinase inhibitor (i.e., p21 and p27) in most of the cell lines tested (Figure 2d and Supplementary Figure 3). Taken together, we believe that the cytotoxicity of CYD-6-17 in RCC cells is mainly mediated through apoptosis and cell cycle arrest.

### The molecular targets of CYD-6-17

**Figure 3:**
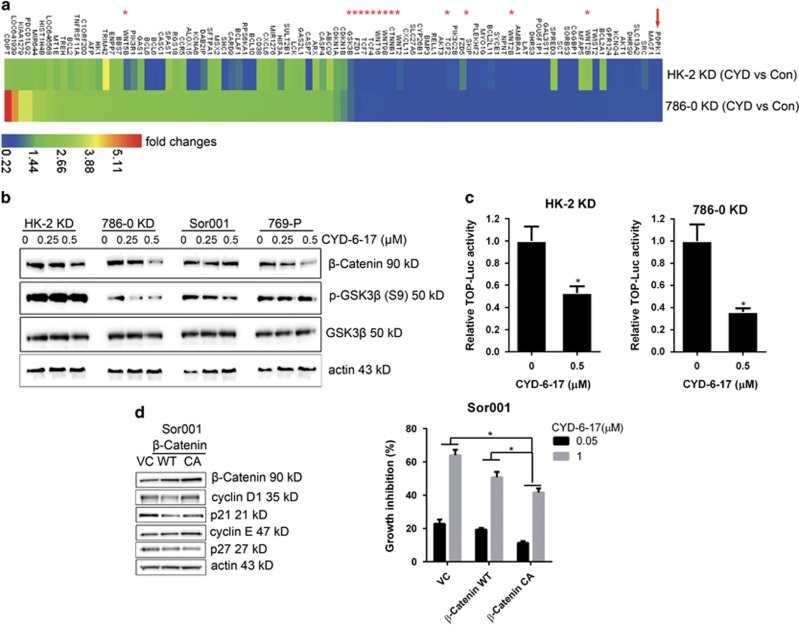
The mechanism of CYD-6-17 in inhibiting Wnt pathway. (**a**) 786-0 KD and HK-2 KD cells were treated with control (Con) or CYD-6-17 for 24 h, the total RNA were subjected to Affymetrix GeneChip cDNA array and heat map were depicted for the relative gene expression (fold changes) normalized with Con. Asterisks indicated Wnt-related effectors. (**b**) Western blot analyses of the expression of GSK3*β* and *β*-catenin in RCC cells treated with CYD-6-17. (**c**) The effect of CYD-6-17 on *β*-catenin-mediated gene transcriptional activity in RCC cells transfected with TOP luciferase reporter gene. After normalizing with Renilla luciferase activity, the relative reporter gene activity in each cell was calculated based on the control. (**d**) Determination of cell cycle regulatory factors in Sor001 cells transfected with vector control, WT, or CA *β*-catenin expression vector for 24 h (left panel) and then treated with CYD-6-17 for 48 h to determine the cell viability using MTT assay (right panel). The growth inhibition rate was calculated based on the control. *P<0.05

To understand the molecular mechanism of CYD-6-17 in drug-resistant RCC cells, microarray analysis was carried out to profile the pattern of mRNA expression after treatment. Several effectors in Wnt-signaling pathway, such as Wnts, FZDs, CTNNB1, and TCFs (Figure 3a) were inhibited in the presence of CYD-6-17. Among them, we further demonstrated that CYD-6-17 could suppress the expression of *β*-catenin mRNA and protein as well as phosphorylated GSK3*β* (p-GSK3*β*, S9) (Figure 3b and Supplementary Figure 4A), which was consistent with decreased *β*-catenin-mediated gene transcriptional activity (Figure 3c). To determine the role of *β*-catenin in cytotoxic effect of CYD-6-17, we established wild-type (WT) or constitutive active (CA) *β*-catenin-expressing cells, and found that both WT and CA *β*-catenin could decrease the expression of p21 and p27 and increase the expression of cyclin E and cyclin D1 (Figure 3d), however, under the treatment of CYD-6-17, WT *β*-catenin failed to rescue CYD-6-17-elicited RCC growth inhibition, CA *β*-catenin was able to antagonize the inhibitory effect of CYD-6-17 (Figure 3d and Supplementary Figure 4B). These data indicated that *β*-catenin is a major target of CYD-6-17 in RCC cells.

### The molecular mechanism of CYD-6-17

**Figure 4:**
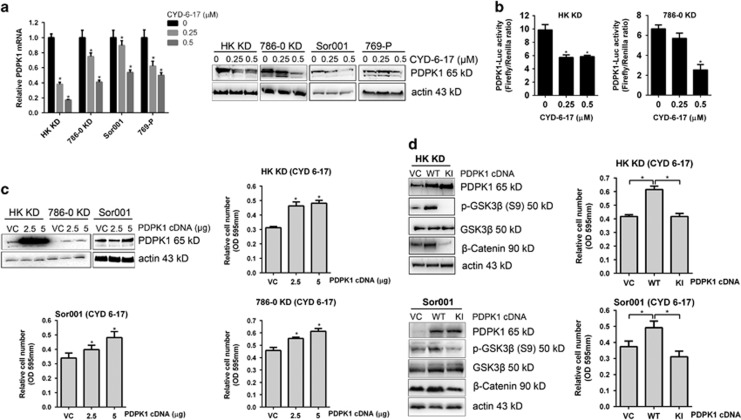
The effect of CYD-6-17 on PDPK1 expression and its role in modulating *β*-catenin expression. (**a**) The expression of PDPK1 mRNA and protein in RCC cells treated with CYD-6-17. (**b**) PDPK1 gene promoter activity determined by luciferase reporter assay in RCC cells treated with CYD-6-17. (**c**) Cells were transfected with different dose of PDPK1 cDNA for 24 h and then treated with CYD-6-17 (0.5 *μ*M) for 48 h. MTT assays were performed to determine cell viability. (**d**) The mechanism of PDPK1 in modulating GSK3*β* activity. HK-2 KD and Sor001 cells were transiently transfected with 2.5 *μ*g of vector control, WT, or KI PDPK1 for 24 h, western blot analyses were performed to determine the expression of PDPK1, total GSK3*β* or phosphorylated GSK3*β*, and *β*-catenin levels (left panel). The relative cell number treated with CYD-6-17 (0.5 *μ*M) for 48 h was determined by MTT assay (right panel). *P<0.05

To further delineate the underlying mechanism of CYD-6-17 in suppressing *β*-catenin expression in RCC cells, we noticed that PDPK1, an AKT activation kinase, was significantly inhibited in CYD-6-17 treatment (Figure 3a). We confirmed this result based on the mRNA and protein expression as well as its promoter activity in a variety of RCC cells (Figures 4a and b). In contrast, overexpression of PDPK1 in RCC cells was able to promote cell survival in the conditions of CYD-6-17 (Figure 4c) by increasing *β*-catenin, cyclin D1, and cyclin E along with decreased p21 and p27 (Supplementary Figure 5A). To demonstrate PDPK1 as a key target of CYD-6-17, both WT and kinase inactive variant (KI, mutated sites K111Q, D205N, D223N) cDNA^10^ were introduced into HK-2 KD and Sor001 cells, and the result indicated that WT PDPK1 but not KI could significantly increase GSK3*β* phosphorylation at S9 (Figure 4d), which is known to be mediated by AKT resulted in the loss of GSK3*β* activity.^11, 12^ As expected, WT PDPK1 transfected cells showed elevated *β*-catenin levels and became resistant to CYD-6-17 treatment (Figure 4d). To further delineate the relationship between PDPK1 and GSK3*β*, PDPK1 and CA GSK3*β* (GSK3 S9A) were co-expressed in HK-2 KD cells and the result clearly showed that the presence of CA GSK3*β* was able to counteract the effect PDPK1 on increasing *β*-catenin protein levels (Supplementary Figure 5B).

**Figure 5:**
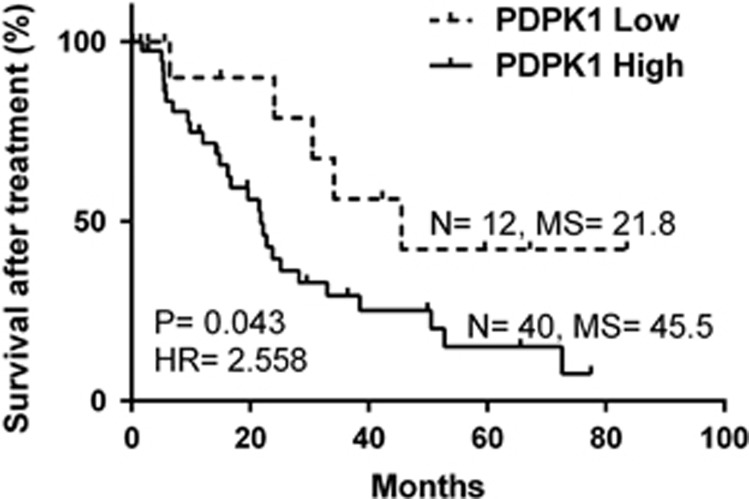
Association of PDPK1 and RCC patient survival who underwent targeted therapies in TCGA data set. PDPK1 mRNA expression data (RNA-Seq) of RCC patient underwent targeted therapies in TCGA was retrieved, and the X-tile was used to generate an optimal cutoff point to dichotomize PDPK1 mRNA level as ‘High' and ‘Low' using a Monte-Carlo *P*-value <0.05. The Kaplan–Meier method (log-rank test) was used to analyze patient survival after treatments. Sample size (N), median survival (MS), and hazard ratio (HR) are depicted

Since PDPK1 is a serine/threonine protein kinase with many known substrates such as AKT,^13^ we therefore determined whether PDPK1-mediated AKT phosphorylation is involved in CYD-6-17 activity. Indeed, CYD-6-17 significantly suppressed AKT phosphorylation and P70S6K1 phosphorylation (Supplementary Figure 6A). Similar to the result of overexpression of PDPK1, the overexpression of CA AKT could rescue RCC cells from CYD-6-17-induced growth inhibition (Supplementary Figure 6B). Noticeably, data analyses of reverse-phase protein array-based protein expression from 212 clinical RCC samples indicated that activated PDPK1 (S241) was positively correlated with PI3K, AKT, GSK3*β*, *β*-catenin, and mTOR/P70S6K1 and negative correlated with cell cycle and apoptotic proteins (Table 1). In addition, Kaplan–Meier survival analysis of RCC patients received targeted therapies (mTOR inhibitors or tyrosine kinase inhibitor, Supplementary Table 1) from TCGA showed that PDPK1 was negatively associated with patient survival after treatments (*P*=0.043, HR=2.558, Figure 5). Thus, we believe PDPK1 plays a critical role in the efficacy of RCC therapy and PDPK1 should be considered as a therapeutic target.

### The *in vivo* effect of CYD-6-17 on of RCC tumor

**Figure 6:**
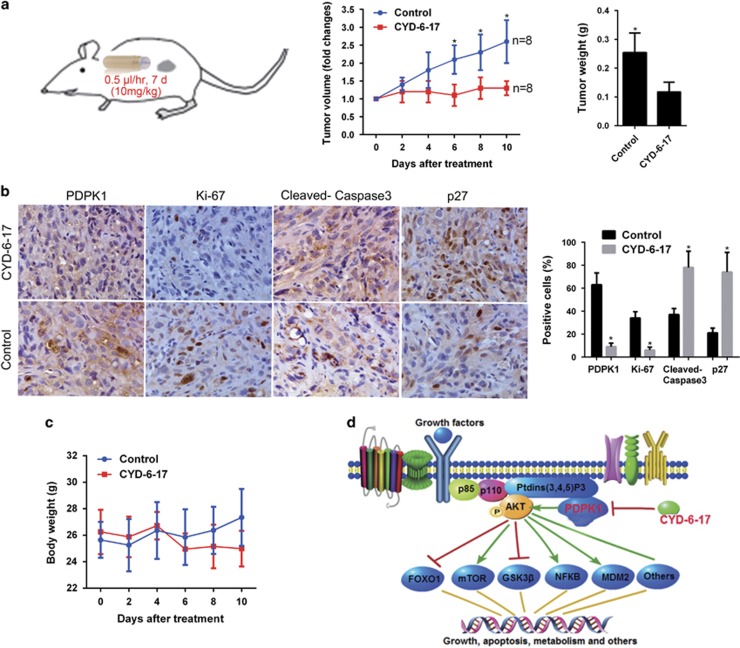
The efficacy of CYD-6-17 in RCC tumor *in vivo*. (**a**) The experimental therapy of CYD-6-17 using ALZET osmotic pumps to deliver 10 mg/kg of drug for 7 days in 786-0 KD subcutaneous xenograft model. Both tumor volume measured by caliper and tumor weight of control or treatment group were depicted. *P<0.05. (**b**) Immunohistochemistry staining of PDPK1, Ki-67 (proliferation marker), cleaved caspase-3 (apoptosis marker) and p27 (cell cycle arrest marker) in RCC tumors. Quantitative data were shown in the right panel. (**c**) The total body weight of animal from control and treatment group. (**d**) A schematic presentation of the mechanism of CYD-6-17 in targeting PDPK1 and its downstream pathways in drug-resistant RCC

We therefore evaluated the antitumor effect of CYD-6-17 using preclinical RCC xenograft models (Figure 6a). The results showed that CYD-6-17 significant decreased tumor volume and weight of RCC tumors compared with the control group. Consistently, CYD-6-17 significantly decreased the expression of PDPK1 and Ki67 but increased both apoptotic and cell cycle markers cleaved caspase-3 and p27 in tumors validated by immunohistochemistry (Figure 6b). In addition, CYD-6-17 did not elicit significant side effect based on overall animal weight (Figure 6c).

## Discussion

RCC is one of the most lethal urologic malignancies with deaths of more than 100 000 per year worldwide.^14^ Surgical treatment remains the standard of care for localized, non-metastatic RCC, however, 1/3 of patients who undergo surgical resection for local disease will develop metastatic disease, and when metastatic, it is largely incurable with a very poor 5-year survival rate.^15^ In general, RCC is insensitive to conventional chemotherapy and radiotherapy.^16^ Although several targeted therapies have improved patient survival, mRCC remains incurable due to the low-response rate and rapid development of drug resistance to these agents. Clearly, there are needs for (1) searching new agents that have different mechanism of actions; (2) identifying key pathways underlying resistance. Therefore, ideas such as targeting key signaling controlling RCC metabolism or cellular immune response have been proven to be promising approaches for RCC.^17, 18, 19^ On the other hand, by delineating the mechanisms of RCC resistant to radiation, we formulated a new strategy of simultaneously targeting HIF-2*α* and SHH-GLI pathways that could significantly sensitize RCC to radiation.^20^ We also found the combination of mTOR and ERK inhibitors could cause a synthetic lethal of drug-resistant RCC.^9^ Based on combination strategy, in this study, we attempt to search new agent with multiple targeting capability.

Recently, natural product has more attention as potential new cancer therapeutics due to its multiple mechanism of action and low side effects.^3^ Oridonin is one of the active ingredient isolated from Chinese medicinal herb *Rabdosia rubescens* and has been shown to have antitumor activities in many cancers, such as esophagus, mammary gland, liver, and prostate cancers.^21, 22, 23^ However, clinical application of oridonin has been limited because it has moderate potency, poor solubility, and bioavailability.^8^ Recently, we have developed new chemical synthesis to generate a variety of nitrogen-enriched oridonin analogs based on structurally diversified diterpenoids.^6^ Among them, CYD-6-17 that is fused at C-1 and C-2 of the A-ring (Figure 1b), has a better potency and aqueous solubility^6^ and no major side effect in animal, exhibited very high potency to RCC cells with IC_50_ at nanomolar range. We recently unveiled molecular mechanism leading to targeted therapeutics resistance in a variety of RCC cell lines;^9^ several have acquired resistant (e.g., ACHN from metastatic RCC and Sor001 from a patient resistant to sorafenib) and the others are genetically induced (such as HK-2 KD and 786-0 KD). By screening these cell lines, we noticed that CYD-6-17 appeared to be very potent with similar IC_50_ at nanomolar range. Interestingly, CYD-6-17 does not damage endothelia, instead, specifically inhibits the growth of endothelia induced by RCC cells. Thus, we believe CYD-6-17 has a good potential clinical application as the second-line therapeutic for drug-resistant RCC.

Despite the large number of studies reporting that oridonin can trigger apoptosis, cell cycle arrest, and autophagy in different neoplastic cell lines, the entire molecular mechanism underlying its multi-targeted effects remains to be elucidated. Several proteins and receptors with which oridonin can directly or indirectly interact have been reported. Among the targets of oridonin, proteases, transcription factors, and kinases (e.g., telomerase, c-Myc, EGFR, p21, NF-κB, Ras, JNK, and p38) have been characterized.^24, 25, 26, 27, 28^ In RCC, we identify a novel target of CYD-6-17, PDPK1, and unveil that GSK3*β*/*β*-catenin is the key downstream effector of PDPK1 in mediating RCC growth. The oncogenic *β*-catenin signaling in RCC has been well documented. Strong intracellular staining of *β*-catenin was associated with the high grade of RCC tumors, which constitute the most aggressive form of the tumors, and stabilization of *β*-catenin is required for proliferation of RCC cells.^29^ Cells in the kidney of transgenic mice overexpressing an activated *β*-catenin transgene exhibited increased proliferation.^30^ These findings imply that targeting *β*-catenin signaling will bring therapeutic benefits for RCC. In the present study, we demonstrate that CYD-6-17 interferes *β*-catenin signaling via directly targeting its upstream regulator PDPK1. PDPK1 is the key kinase transducing signals from PI3Ks to AKT, which phosphorylates and activates AKT.^13^ As major effectors downstream of receptor tyrosine kinases and G protein-coupled receptors, PI3Ks transduce signals from various growth factors and cytokines into intracellular messages by generating phospholipids, which activate the protein kinase AKT, eliciting a broad range of downstream signaling events, such as GSK3*β*, mTOR, FOXO1, NF-κB, MDM2, and BAD^13^ (Figure 6d). PI3K signaling pathway is the master signaling controlling RCC development and progression.^31^ Inhibitors that target PI3K isoforms and other major nodes in the pathway, including AKT and mTOR, reach certain clinical benefits, however, major issues remain, such as limited efficacy or development of resistance to therapies. Our previous studies^9, 20^ demonstrated that inhibitors of AKT or mTOR only exhibited little or moderate efficacy to RCC cells. A novel inhibitor of AKT1–PDPK1 interaction is reported to efficiently restrict tumor growth in prostate cancer.^32^ Here we show that PDPK1 is significantly associated with RCC patient's survival after treatment, notably, we demonstrate that CYD-6-17 can target PDPK1 and its downstream AKT, subsequently, effectively inhibits RCC cell growth, thus discover a novel anticancer target and provided a promising therapeutic strategy.

## Materials and Methods

### Cell culture

769-P, 786-0, and HK-2 cells were maintained in RPMI-1640 medium containing 10% fetal bovine serum. ACHN, A498, OSRC-2, and HEK293 cells were maintained in DMEM containing 10% fetal bovine serum. Several drug-resistant RCC lines, such as HK-2 KD and 786-0 KD cells, described in our recent publication^9^ as well as Sor001 cell derived from mRCC patients resistant to sorafenib were maintained in RPMI-1640 medium containing 10% fetal bovine serum. Human umbilical vein endothelial cell (HUVEC) cell was maintained in endothelial cell medium (ScienceCell, Carlsbad, CA, USA) containing 5% fetal bovine serum and 1% endothelial cell growth supplement (ScienceCell). For co-culture assays, HUVEC (3 × 10^4^ cells/well) was plated in the bottom chamber and 786-0 (1.5 × 10^4^ cells/well) was plated in the upper transwell chamber (0.4 *μ*m pore size, Fisher Scientific, Waltham, MA, USA). CYD-6-17 or solvent control was added to the co-culture medium, and cell growth of HUVEC was determined at 48 h after plating by crystal violent staining with the removal of 786-0 cells.

### Cell proliferation

Cells were re-suspended in appropriate medium and cultured in 96-well plates at the density of 1500 cells/well overnight. Cells were then treated with CYD-6-17 for 48 h and cell viability was assessed using a 3-(4,5-dimethylthiazol-2-yl)-2,5-diphenyltetrazolium bromide (MTT) assay (Roche, Indianapolis, IN, USA) according to the manufacturer's instructions.

### Cell apoptosis

Cells were treated with various concentrations of CYD-6-17 for 24 h. Cells were then collected, washed with PBS, and then subjected to annexin V and PI staining using an annexin V-FITC apoptosis detection kit (Invitrogen, Carlsbad, CA, USA) and immediately analyzed by flow cytometry (FACSCalibur, BD Biosciences, Franklin Lakes, NJ, USA).

### Constructs and transfection

PDPK1 WT and kinase inactive variant (KI, mutated sites K111Q, D205N, D223N) cDNA cloned into pcDNA3-Myc vectors were gifts from Dr. Melanie Cobb in University of Texas Southwestern Medical Center. *β*-Catenin WT cDNA and the CA of *β*-catenin and GSK-3*β* (GSK-3*β* CA) cDNA were all cloned into pcDNA3 vectors. For transfections, 3 × 10^5^ cells were seeded in six-well plate with 60% confluence prior to transfection, and cDNA were transfected into cells by using Lipofecamine LTX PLUS (Invitrogen) according to the manufacturer's instructions.

### Luciferase assay

Cells seeded in 24-well plates were transfected with either 1 *μ*g *β*-catenin reporter (TOP-Luc) or PDPK1 promoter firefly luciferase constructs, and 2 ng Renilla luciferase construct as internal control for 48 h. Luciferase activity was measured using dual luciferase assay kit according the manufacturer's protocol (Promega, Madison, WI, USA).

### cDNA microarray

RAN was isolated from HK-2 KD and 786-0 KD treated with control (Con) or CYD-6-17 for 24 h, and then applied to Affymetrix GeneChip Array at the UTSW Genomics and Microarray Core facility. Biological duplicate samples were tested and arrays were visualized and analyzed using R Project.

### Real-time reverse-transcription polymerase chain reaction

Cell total RNA was extracted with RNeasy mini kit (Qiagen, Valencia, CA, USA) and 2 *μ*g RNA was reverse transcribed with VILO cDNA Synthesis Kit (Invitrogen). Real-time reverse-transcription polymerase chain reaction analysis was carried out using SYBR Green qPCR Supermix kit (Invitrogen) in iCycler thermal cycler (Bio-Rad, Hercules, CA, USA). The relative level of PDPK1 mRNA expression was determined by normalizing to 18S rRNA. Primer sequences used for PDPK1 were: 5′-GAACAGACTGGCCTC CTACTT G-3′ (forward) and 5′-TGACAACTAAAGGAGGATGTGG-3′ (reversed).

### Western blot

Cells were collected and lysed using ice-cold lysis buffer (150 mM NaCl, 1% Triton X-100, 0.5% sodium deoxycholate, 0.1% SDS, 50 mM Tri (pH 8.0), protease inhibitor cocktail (Roche)) for 45 min. Lysates were centrifuged at 13 000 rpm for 10 min at 4 °C and supernatant was collected. Equivalent amounts of protein were separated on 4–12% gradient NuPAGE Bis-Tris Gels (Invitrogen) and transferred to nitrocellulose membranes. Membranes were blocked in TBS-Tween20 containing 3% nonfat dry milk (w/v) for 1 h and then incubated with primary antibodies overnight at 4 °C. Appropriate secondary antibodies conjugated with horseradish peroxidase and signals were then detected by enhanced chemiluminescence (Pierce, Rockford, WI, USA).

### Immunohistochemistry staining

Formalin-fixed, paraffin-embedded sections were deparaffinized, rehydrated, and subjected to heat-induced antigens retrieval. Sections were blocked with goat serum, incubated with primary antibodies, and developed with 3,3′-diaminobenzidine chromogen followed by counter staining with hematoxylin. Primary antibodies used were as follow: rabbit anti-PDPK1 (Ab-241) was from Sigma (St. Louis, MO, USA), mouse anti-Ki67 (556026) was from BD Biosciences (San Jose, CA, USA), cleaved caspase-3 (D3E9) and p27 (D69C12) were from Cell Signaling Technology (Danvers, MA, USA).

### Animal experiment

All experimental procedures were approved by the Institutional Animal Care and Use Committee. For drug treatment, 2 × 10^6^ of 786-0 KD cells were subcutaneously injected into mice. When the tumor volume reached at 50 mm^3^, animals were randomly grouped. ALZET osmotic pump (DURECT Corporation, Cupertino, CA, USA) containing vehicle control or CYD-6-17 was implanted subcutaneously into each mouse nearby the tumors. Tumor volume was recorded very other day and calculated by using the ellipsoid formula. At the end of experiment, mice were killed and tumor tissues were dissected for pathological study.

### Bioinformatics and statistical analysis

Reverse phase protein array-based protein expression data of 212 RCC samples, PDPK1 mRNA expression data of RCC samples and the clinical annotation data were retrieved from the TCGA data portal^33^ (http://cancergenome.nih.gov/; update to 4 February 2014). The X-tile which is a bioinformatics tool for biomarker assessment and outcome-based cut-point optimization^34^ was used to generate an optimal cutoff point to dichotomize PDPK1 mRNA level as ‘High' and ‘Low' using a Monte-Carlo *P*-value <0.05. Kaplan–Meier method (log-rank test) was used to analyze patient survival. All the *in vitro* experiment data was presented as the mean±S.E.M. from at least three independent experiments and the differences between two groups were compared by the Student's *t*-test. All statistical analyses were performed by GraphPad Prism 6 and SPSS16.0.

## Figures and Tables

**Table 1:** Correlation between PDPK1 (S241) protein phosphorylation and PI3K, AKT, GSK3*β*, *β*-catenin, mTOR pathway, regulators of cell cycle, and apoptosis in RCC patients from TCGA data set

	**PDPK1 (S241)**				
	**Pearson's R value**	**95% confidence interval**	**R squared**	**P value**	**Number of samples**
PI3K-p85	0.5229	0.4176 to 0.6144	0.2734	< 0.0001	212
PI3K-p110*α*	0.445	0.3301 to 0.5470	0.198	< 0.0001	212
p38 (T180/Y182)	0.2917	0.1634 to 0.4103	0.0851	< 0.0001	212
AKT	0.5779	0.4805 to 0.6611	0.3339	< 0.0001	212
AKT (S473)	0.3693	0.2468 to 0.4801	0.1364	< 0.0001	212
AKT (T308)	0.2968	0.1688 to 0.4149	0.08808	< 0.0001	212
DVL3	0.2751	0.1458 to 0.3952	0.07569	< 0.0001	212
GSK3*αβ* (S21/S9)	0.5495	0.4479 to 0.6371	0.3019	< 0.0001	212
GSK3 (S9)	0.4542	0.3402 to 0.5550	0.2063	< 0.0001	212
*β*-Catenin	0.3572	0.2337 to 0.4694	0.1276	< 0.0001	212
mTOR	0.6788	0.5988 to 0.7454	0.4608	< 0.0001	212
mTOR (S2448)	0.3745	0.2525 to 0.4848	0.1402	< 0.0001	212
p70S6K	0.4868	0.3768 to 0.5833	0.237	< 0.0001	212
p70S6K (T389)	0.3	0.1722 to 0.4178	0.08999	< 0.0001	212
eIF4E	0.1591	0.02492 to 0.2877	0.02532	0.0204	212
4E-BP1	−0.4188	−0.5240 to −0.3011	0.1754	< 0.0001	212
4E-BP1 (T70)	−0.3866	−0.4956 to −0.2657	0.1495	< 0.0001	212
p21	−0.2406	−0.3636 to −0.1094	0.05789	0.0004	212
p53	−0.4344	−0.5377 to −0.3183	0.1887	< 0.0001	212
Bak	−0.4463	−0.5481 to −0.3315	0.1992	< 0.0001	212
Bid	−0.5781	−0.6614 to −0.4808	0.3342	< 0.0001	212
Cleaved caspase3	−0.4742	−0.5723 to −0.3626	0.2248	< 0.0001	212
Cleaved caspase7	−0.5369	−0.6264 to −0.4336	0.2883	< 0.0001	212
Caspase8	−0.3591	−0.4710 to −0.2357	0.1289	< 0.0001	212
Cleaved caspase9	−0.2215	−0.3460 to −0.08947	0.04908	0.0012	212
Cleaved PARP	−0.4993	−0.5941 to −0.3908	0.2493	< 0.0001	212
