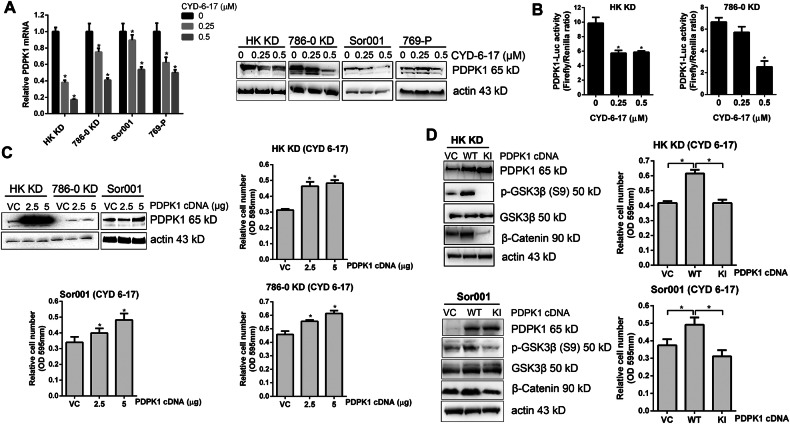# Correction: Targeting 3-phosphoinositide-dependent protein kinase 1 associated with drug-resistant renal cell carcinoma using new oridonin analogs

**DOI:** 10.1038/s41419-026-08828-5

**Published:** 2026-06-08

**Authors:** Jiancheng Zhou, Eun-Jin Yun, Wei Chen, Ye Ding, Kaijie Wu, Bin Wang, Chunyong Ding, Elizabeth Hernandez, John Santoyo, Rey-Chen Pong, Haiying Chen, Dalin He, Jia Zhou, Jer-Tsong Hsieh

**Affiliations:** 1 Department of Urology, Shaanxi Provincial People’s Hospital, Xi’an, 710068 Shaanxi P.R. China; 2 Department of Urology, University of Texas Southwestern Medical Center, Dallas, TX 75390 USA; 3 Department of Urology, The First Affiliated Hospital, Medical School of Xi’an Jiaotong University, Xi’an, 710061 China; 4 Department of Pharmacology and Toxicology, University of Texas Medical Branch, Galveston, TX 77555 USA; 5 Institute of Urology, Medical School of Xi’an Jiaotong University, Xi’an, 710061 China

Correction to: *Cell Death & Disease* 10.1038/cddis.2017.121, published online 23 March 2017

During a recent internal review, we identified an error in Figure 4D (lower panel) regarding the Actin protein band for the Soor1 cell line. Specifically, an incorrect western blot image was inadvertently included during figure assembly due to the misplacement of raw image files. The corrected Figure 4D is showing the correct Actin loading control.

**Original Figure 4** 
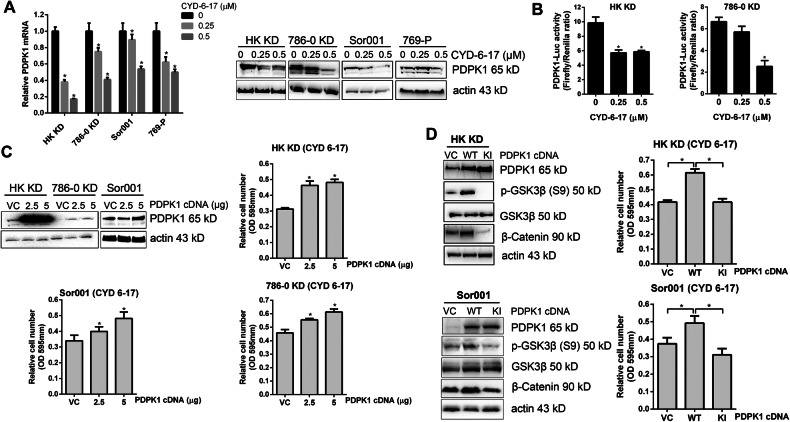


**Corrected Figure 4**