# Supplementary material for: Targeting 3-phosphoinositide-dependent protein kinase 1 associated with drug-resistant renal cell carcinoma using new oridonin analogs
Source: Cell Death Dis. 2017 Mar 23;8(3):e2701–. doi: 10.1038/cddis.2017.121 (PMC5386527; doi:10.1038/cddis.2017.121)
Supplement: Supplementary Information [file cddis2017121x1.pdf]

Supplemental Figure 1. The effect of CYD-6-17 or Oridonin on the growth of drug resistant cells. The effects of CYD-6-17 or Oridonin on growth of resistant cells (HK KD, 786-0 KD and Sor001) were determined by MTT assay. * p< 0.05.

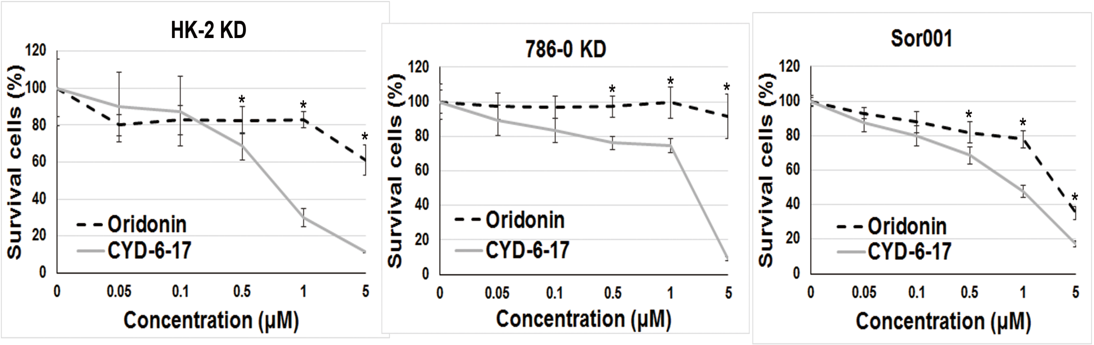

Supplemental Figure 2. The comparison of the effect of CYD-6-17 on the growth of parental cells to drug resistant cells. A. The effects of CYD-6-17 on growth of parental cells (HK Con, 786-0 Con) and other RCC cells were determined by MTT assay. Cell growth inhibition rate was calculated by normalization with the vehicle group (0 μM of CYD-6-17). **B.** Comparison of the potency of CYD-6-17 on the growth of parental cells (Con) and drug resistant cells (KD). * p< 0.05.

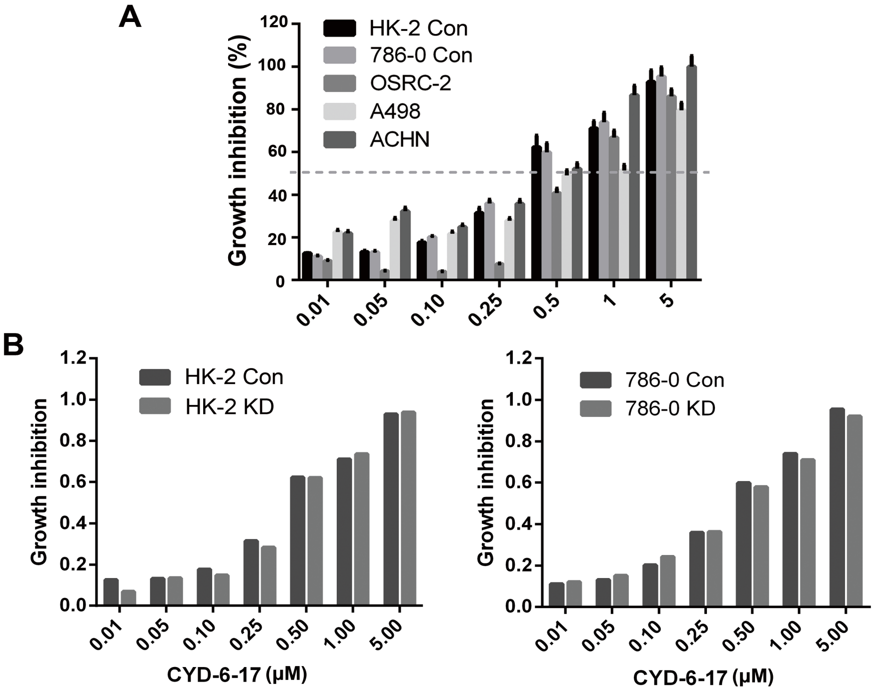

Supplemental Figure 3. Quantification of the expression of a panel of cell cycle regulatory factors in RCC cells treated with different concentrations of CYD-6-17.

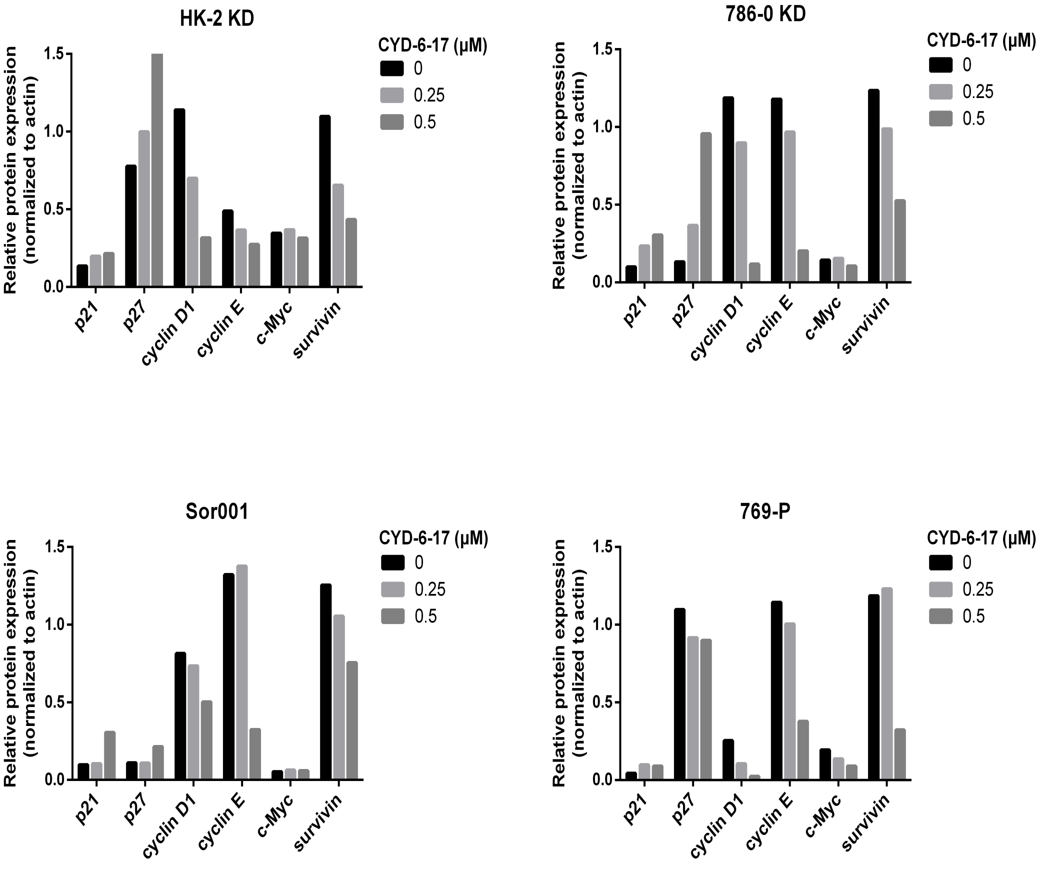

Supplemental Figure 4. The role of β-Catenin in the growth inhibitory effect of CYD-6-17 on RCC cells. A. Real time-PCR detecting the expression of β-Catenin mRNA in cells treated with CYD-6-17 for 48 hr. **B.** HK-2 KD cells were transiently co-transfected with wild type (WT) or constitutive activated (CA) for β-Catenin 24 hr and then treated with CYD-6-17 (0.5 μM) for 48 hr. Western blot analysis was performed to determine the expression of β-Catenin levels, and MTT assays were performed to determine cell viability. * $p < 0.05$.

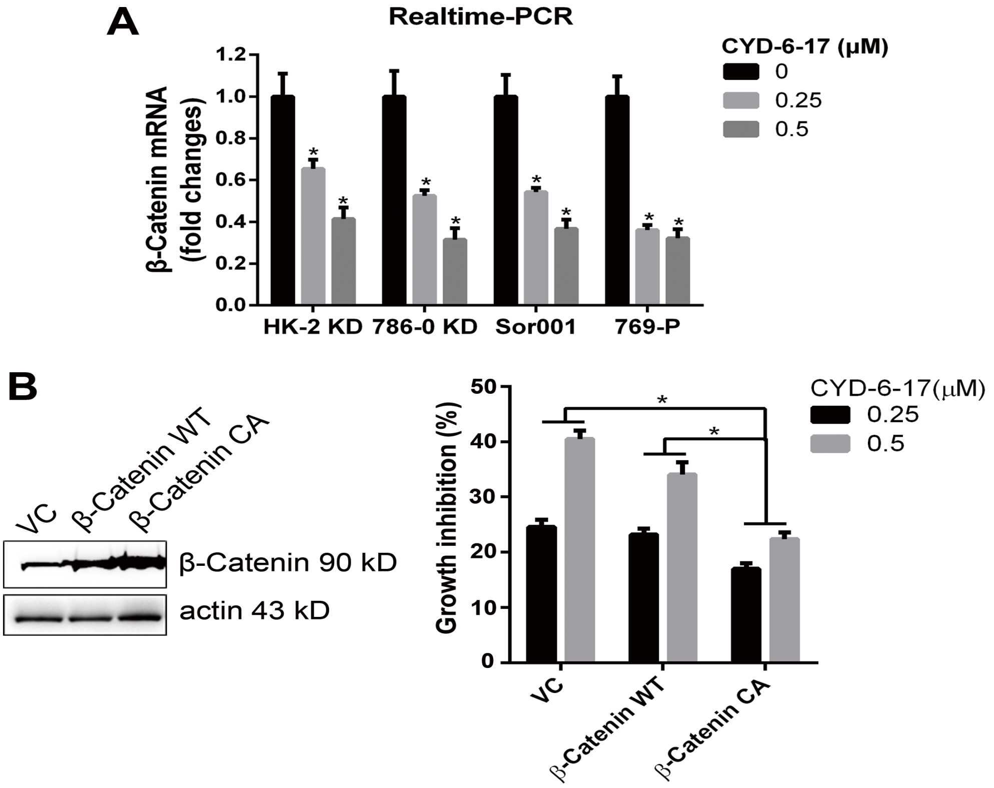

Supplemental Figure 5. The relationship between GSK3β and PDPK1 in modulating β-Catenin protein levels. A. Sor001 cells were transiently co-transfected with different dose of PDPK1 cDNA for 48 hr, western blot analyses were performed to determine the expression of PDPK1, β-Catenin and cell-cycle related markers. B.HK-2 KD cells were transiently co-transfected with constitutive activated (CA) GSK3β and wild type (WT) PDPK1 for 48 hr. Western blot analyses were performed to determine the expression of PDPK1, GSK3β, and β-Catenin levels.

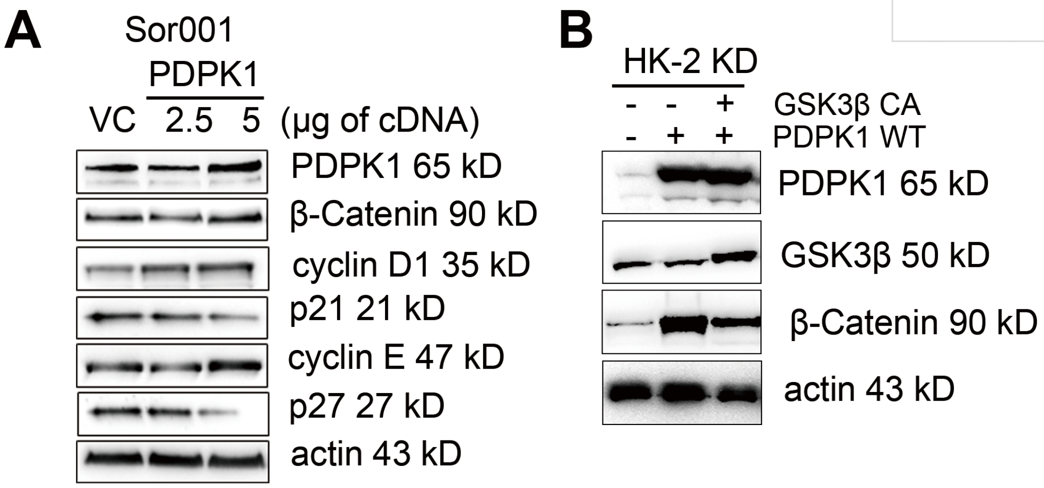

Supplemental Figure 6. The role of AKT in the growth inhibitory effect of CYD-6-17 on RCC cells. A. Western blot analyses of the expression of total AKT, activated AKT (p-AKT), total P70S6K1 and phosphorylated P70S6K1 in HK-2 KD cells treated with CYD-6-17. **B.** HK-2 KD cells were transfected with VC or CA AKT for 24 hr. Western blot analyses of the expression of AKT protein (Let panel) and MTT assay of determining cell viabilities treated with control or CYD-6-17 (Right panel). * $p< 0.05$.

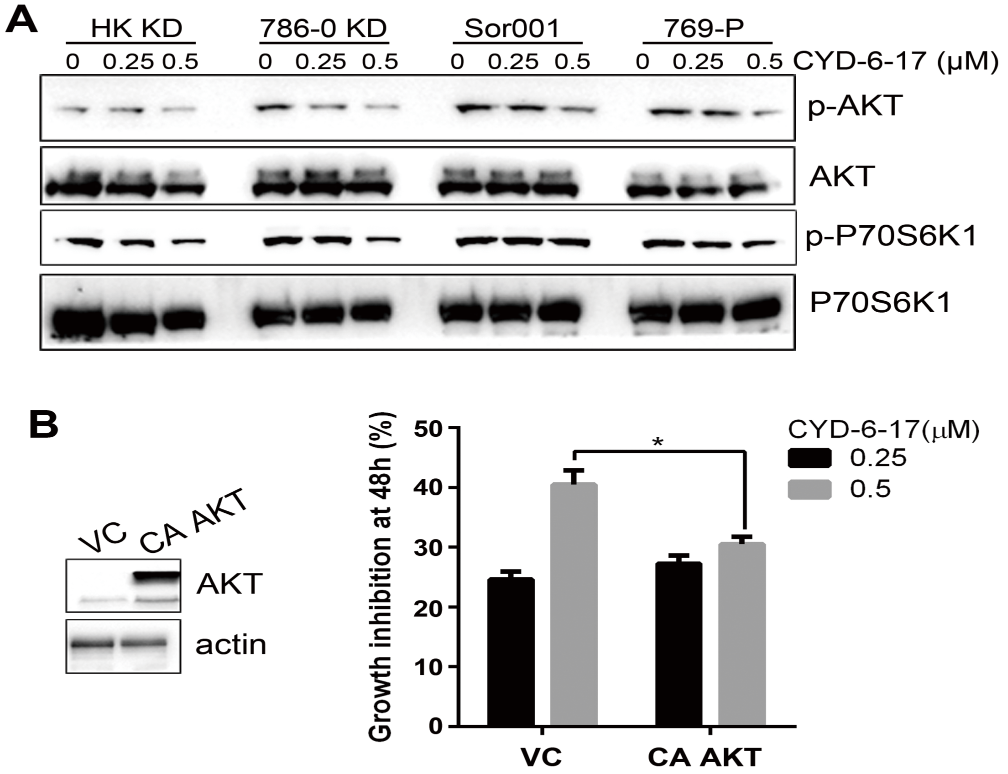

Supplemental Table 1. RCC patients received targeted therapies in TCGA dataset.

bcr_patient_barcode	drug_name	vital_status 1-dead; 0-alive	followup time after treatments (days)	relative PDPK1 mRNA level (RNA-Seq)
TCGA-CZ-5469	Sunitinib	1	723	767.6119
TCGA-BP-4165	Sunitinib	0	450	776.6397
TCGA-CJ-6033	Avastin Gemcitabine INF	1	192	805.9682
TCGA-B0-5107	Sunitinib	1	916	809.3443
TCGA-B8-4153	Pazopanib	0	167	843.4227
TCGA-CJ-5676	Pazopanib	0	2507	965.7773
TCGA-CJ-4881	Temsirolimus	0	83	978.5176
TCGA-CJ-4890	Sorafenib Sunitinib Tipifarnib Interferon	0	2016	987.3751
TCGA-CJ-4900	Tarceva Avastin	1	1366	987.3751
TCGA-BP-4354	Sunitinib Sorafenib Temsirolimus Gefitinil	1	1024	994.4899
TCGA-CJ-4869	Nexavaar Sunitinib	0	1787	996.4974
TCGA-CJ-4875	Nexavaar	0	1270	1038.5024
TCGA-CJ-4638	Gemictibine 5-Flu IL-2 Avastin	1	361	1039.7213
TCGA-CJ-4895	Tarceva Avastin Avastin	1	1155	1054.1001
TCGA-CJ-4888	Sunitinib	1	991	1060.566
TCGA-CJ-4868	Avastin Proleukin Gemcitabine	1	589	1063.8491
TCGA-B0-5694	Pazopanib	1	52	1077.2664
TCGA-B0-4837	Tyrosine kinase inhibitor	1	159	1109.796
TCGA-CZ-4860	Sorafenib	1	152	1165.3465
TCGA-CJ-4644	Intron A Capecitabine Avastin Tarceva	1	174	1169.9194
TCGA-BP-4985	Sunitinib	1	645	1174.2641
TCGA-B8-5162	Sunitinib	0	79	1186.3214
TCGA-BP-5178	Sorafenib	1	1516	1188.4795
TCGA-CJ-4637	Temsirolimus Roferon-A Intron A Sunitini	1	2180	1198.7575
TCGA-CJ-4904	Nexavaar	0	1497	1205.0755
TCGA-BP-5009	Sunitinib Everolimus Bevacizumb Pazopa	1	754	1218.1231
TCGA-BP-4787	Sunitinib Sorafenib Temsirolimus	1	427	1230.6798
TCGA-CZ-4861	Sorafenib - Nexavar	1	488	1252.2936
TCGA-B0-5115	Afinitor (Everolimus)	0	586	1256.3873

TCGA-BP-4804	Sunitinib	0	171	1274.4479
TCGA-BP-4169	Interferon Axitinib	1	665	1275.8655
TCGA-BP-4329	Interferon Temsirolimus	1	655	1293.7959
TCGA-CZ-5464	Pazopanib Sunitinib	0	85	1384.131
TCGA-CJ-4871	Alpha Interferon	0	2325	1393.9394
TCGA-CW-5590	Sunitinib	1	847	1453.2259
TCGA-CZ-5461	Sunitinib	1	285	1497.6097
TCGA-BP-4974	Sunitinib Sorafenib Gefitinib	1	164	1507.8756
TCGA-CZ-5454	Sunitinib	1	685	1530.7013
TCGA-BP-4161	Sunitinib	0	6	1540.4033
TCGA-B0-5094	Torisel	1	209	1590.1798
TCGA-AK-3436	Sunitinib	0	1968	1601.1777
TCGA-BP-4338	Sunitinib Sorafernib Everolimus	0	1094	1665.9879
TCGA-BP-5189	Temsirolimus Bevacizumb	1	162	1686.364
TCGA-BP-4342	Sorafenib	1	503	1741.1414
TCGA-CZ-5462	Sunitinib	1	296	1859.826
TCGA-CJ-5680	Avastin IL-2 Tarceva	1	715	1906.0533
TCGA-CW-5585	Sunitinib	0	46	1917.2368
TCGA-CJ-6028	Interferon Sunitinib Sorafenib	1	1584	1982.8386
TCGA-CJ-5681	IL-2 Gemcitabine Avastin	1	446	1983.0564
TCGA-CZ-5456	Pazopanib	0	434	2055.6603
TCGA-B2-5639	Torisel	0	343	2124.3705
TCGA-A3-3317	Sorafenib	0	886	2600.483
